# Long-term pulmonary outcome of children with congenital diaphragmatic hernia: functional lung MRI using matrix-pencil decomposition enables side-specific assessment of lung function

**DOI:** 10.1007/s00330-023-10395-8

**Published:** 2023-11-20

**Authors:** Carmen Streibel, C. Corin Willers, Grzegorz Bauman, Orso Pusterla, Oliver Bieri, Marion Curdy, Matthias Horn, Carmen Casaulta, Steffen Berger, Gabriela Marta Dekany, Elisabeth Kieninger, Andreas Bartenstein, Philipp Latzin

**Affiliations:** 1 Division of Paediatric Respiratory Medicine and Allergology, Department of Paediatrics Inselspital, Bern University Hospital, University of Bern, Bern, Switzerland; 2 Graduate School for Health Sciences, University of Bern, Bern, Switzerland; 3 Department of Paediatrics, Kantonsspital Aarau, Aarau, Switzerland; 4 Department of Radiology, Division of Radiological Physics, University of Basel Hospital, Basel, Switzerland; 5 Department of Biomedical Engineering, University of Basel, Allschwil, Switzerland; 6 Department of Paediatric Surgery, Inselspital, Bern University Hospital, Bern, Switzerland

**Keywords:** Congenital diaphragmatic hernias, Children, Pulmonary function test, Lung, Functional magnetic resonance imaging

## Abstract

**Objectives:**

In patients with congenital diaphragmatic hernia (CDH) the exact functional outcome of the affected lung side is still unknown, mainly due to the lack of spatially resolved diagnostic tools. Functional matrix-pencil decomposition (MP-) lung MRI fills this gap as it measures side-specific ventilation and perfusion. We aimed to assess the overall and side-specific pulmonary long-term outcomes of patients with CDH using lung function tests and MP-MRI.

**Methods:**

Thirteen school-aged children with CDH (seven with small and six with large defect-sized CDH, defined as > 50% of the chest wall circumference being devoid of diaphragm tissue) and thirteen healthy matched controls underwent spirometry, multiple-breath washout, and MP-MRI. The main outcomes were forced expiratory volume in 1 second (FEV_1_), lung clearance index (LCI_2.5_), ventilation defect percentage (VDP), and perfusion defect percentage (QDP).

**Results:**

Patients with a large CDH showed significantly reduced overall lung function compared to healthy controls (mean difference [95%-CI_adjusted_]: FEV_1_ (z-score) −4.26 [−5.61, −2.92], FVC (z-score) −3.97 [−5.68, −2.26], LCI_2.5_ (TO) 1.12 [0.47, 1.76], VDP (%) 8.59 [3.58, 13.60], QDP (%) 17.22 [13.16, 21.27]) and to patients with a small CDH. Side-specific examination by MP-MRI revealed particularly reduced ipsilateral ventilation and perfusion in patients with a large CDH (mean difference to contralateral side [95%-CI_adjusted_]: VDP (%) 14.80 [10.50, 19.00], QDP (%) 23.50 [1.75, 45.20]).

**Conclusions:**

Data indicate impaired overall lung function with particular limitation of the ipsilateral side in patients with a large CDH. MP-MRI is a promising tool to provide valuable side-specific functional information in the follow-up of patients with CDH.

**Clinical relevance statement:**

In patients with congenital diaphragmatic hernia, easily applicable MP-MRI allows specific examination of the lung side affected by the hernia and provides valuable information on ventilation and perfusion with implications for clinical practice, making it a promising tool for routine follow-up.

**Key Points:**

*• Functional matrix pencil decomposition (MP) MRI data from a small sample indicate reduced ipsilateral pulmonary ventilation and perfusion in children with large congenital diaphragmatic hernia (CDH).*

*• Easily applicable pencil decomposition MRI provides valuable side-specific diagnostic information on lung ventilation and perfusion. This is a clear advantage over conventional lung function tests, helping to comprehensively follow up patients with congenital diaphragmatic hernia and monitor therapy effects.*

**Supplementary Information:**

The online version contains supplementary material available at 10.1007/s00330-023-10395-8.

## Introduction

Advanced therapeutic opportunities and standardized treatment protocols in the acute care of patients with congenital diaphragmatic hernia (CDH) have remarkably increased overall survival rate [1, 2]. Accordingly, a major current challenge is to monitor and prevent chronic lung disease [3] in these patients. Long-term follow-up is especially recommended [4] in severe cases such as in patients with a large initial defect size demanding a patch repair [5–7], requirement of extracorporeal membrane oxygenation (ECMO) after birth, or need of respiratory support for more than 30 days [6, 8–10]. In addition, a standardised classification scheme for the classification of the initial diaphragmatic defect based on its size has been established: (A) defect entirely surrounded by muscle, (B) < 50% or (C) > 50% of the chest wall circumference is devoid of diaphragm tissue, (D) complete or near complete absence of the diaphragm [11].

To date, lung function follow-up of patients with CDH is done by lung function tests such as spirometry and body plethysmography, providing outcomes of the entire lung. However, as these are breathing tests, information on the side differentiation and the functionality of the peripheral lung tissue is lacking. Long-term studies in patients with CDH using lung function tests or assessing pulmonary symptoms and physical performance capacity have so far shown controversial results [3, 12–17].

Functional imaging of the lung allows for assessing the structure and function of the regional tissue and therefore to examine the lung side affected by the hernia separately. Several studies using different approaches such as ventilation (V) perfusion (Q) scintigraphy [18–21], hyperpolarized 3He-MRI [22], and dynamic contrast-enhanced (DCE) MRI [23–26] showed in accordance with morphological studies [27] a persistent reduction of perfusion and less subdivided, enlarged alveoli in the lung ipsilateral of the CDH. The disadvantage of all the above-mentioned techniques is the need for specialized set-ups including hyperpolarization equipment/infrastructure and/or contrast agent.

Unlike these techniques, non-contrast dynamic MRI approaches allow for the assessment of regional ventilation and perfusion through advanced computational analysis of imaging data and without patient exposure to any ionizing radiation, the need for contrast agents, or specific breathing manoeuvers [28–31]. The easy set-up of standard clinical MRI scanners and high feasibility even in young children [32, 33] make these techniques very attractive for use in pediatrics in various diseases [34–37]. To the best of our knowledge, none of them has yet been used in a study of patients with CDH. Matrix-pencil decomposition (MP-)MRI is a promising, very robust approach ensuring high temporal in addition to high spatial resolution by using ultra-fast sequences with highly accelerated parallel imaging [28, 29, 38].

Thus, with this study, we want to investigate the long-term pulmonary outcome of patients with CDH overall and side-specific by using lung function tests and MP-MRI parameters.

## Methods

### Study design and study population

This retrospective observational single-center study was conducted between 05/2017 and 10/2021 at the Children’s University Hospital of Bern, Switzerland. During this period, we recruited all school-aged patients with a CDH diagnosis at our center. They received a standardized call-in for follow-up and were then asked to participate in the study (regardless of symptom severity). In the study participants, morpho-functional lung imaging via MP-MRI was performed in addition to physical examination and assessment of lung function. Only one patient declined to participate in the study. We assigned the patients with CDH to two groups: (i) patients with CDH with an initial defect of size category A and B closed with non-resorbable sutures of the muscle edges and thoracic wall without any other foreign material (“small CDH”), (ii) patients with CDH with an initial defect of size category C and D, where a dome-shaped Gore-Tex patch or a muscle flap was sutured in to replace the absent diaphragm (“large CDH”). A control group was composed of age and sex-matched healthy children. The study was approved by the Ethics Committee of Bern, Switzerland (ID 2017-00088). All parents or caregivers and patients ≥ 14 years old gave informed written consent to participation in the study.

### Data assessment

Study participants attended lung function tests and MP-MRI on the same day in the following order: (i) nitrogen multiple breath washout (N_2_MBW) measurement, (ii) spirometry/ body plethysmography, and (iii) morpho-functional MP-MRI.

### Lung function measurement

#### Spirometry and body plethysmography

Baseline lung function was assessed by spirometry (Jaeger MasterScreen Body plethysmography, CareFusion). Measurements were performed according to ERS/ATS guidelines [39]. Forced expiratory volume at one second (FEV_1_), forced vital capacity (FVC), the ratio of FEV_1_ over FVC (Tiffeneau index: FEV_1_/FVC), the total lung capacity (TLC), and the ratio of residual volume over total lung capacity (RV/TLC) were the primary outcomes. As raw values are strongly dependent on sex, age, height, and ethnicity of the subject, absolute values were converted to z-scores to allow further inter-individual comparisons as recommended by the European Respiratory Society and American Thoracic Society using the provided reference equations [40]. The lower limit of normal (LLN) was defined as −1.64 z-scores [40].

#### Multiple-breath wash-out

N_2_MBW measurements were performed to assess lung ventilation homogeneity. General conditions were set according to guidelines [41] and raw data was processed using the manufacturer’s software (Spiroware V 3.2.1, Eco Medics AG; data reloaded with Spiroware V 3.3.1) [42]. The main outcome was the lung clearance index (LCI_2.5_) which was assessed in original units (turnover, TO) required to lower tracer gas concentration to 2.5% of the initial value. We applied systematic quality control on all N_2_MBW trials [43]. Per patient, we calculated the mean LCI_2.5_ value of at least two acceptable trials to use in further analysis. An LCI_2.5_ value of 7.1 TO was defined as the upper limit of normal (ULN) [44].

### MP-MRI data acquisition and evaluation

MRI examinations including structural and functional scans were performed on a clinical 1.5T whole-body scanner (MAGNETOM Aera, Siemens Healthineers). For functional scans a multi-slice 2D time-resolved ultra-fast balanced steady-state free precession (uf-bSSFP) pulse sequence was used, i.e. the entire chest volume was covered from posterior to anterior with around 8 to 14 coronal slices, and a voxel size of 3.3 mm × 3.3 mm × 12 mm in supine position during free tidal breathing [45]. At each slice location, 150 images were sequentially acquired for approximately 50 seconds with a frame rate of 3.3 images per second (110 ms acquisition time per single image and 190-ms interval between consecutive images) [45]. The image series acquired was registered to a fixed baseline image in the mid-respiratory state to compensate for respiratory motion [46] and the lung parenchyma was segmented automatically [47]. Data were further processed with the matrix pencil (MP) algorithm [28]: Voxel-wise spectral analysis of the amplitudes of periodic lung parenchyma signal intensity modulations caused by respiration (frequency corresponding to respiratory rate) and pulsatile blood flow (frequency corresponding to pulse rate) was used to calculate quantitative ventilation and perfusion maps of the lung [28, 38]. Lung regions with the fractional ventilation or perfusion amplitude below 0.70 of the median of all pixels inside a local region of interest (segmented lung area on the corresponding coronal slice) were considered to show impaired fractional ventilation or impaired perfusion respectively [28, 38].

The main outcomes were ventilation defect percentage (VDP) and perfusion defect percentage (QDP), which equal the relative amount of lung volume with impaired fractional ventilation resp. relative perfusion [28, 38]. Further, the relative volume of regions with matched defects in perfusion and ventilation (VQD_match_) was quantified. The homogeneity of defect distribution for ventilation and perfusion was assessed by the defect distribution index DDI (DDI_V(Ventilation)_ and DDI_Q(Perfusion)_, resp.) [48]. The DDI increases with the defect areas being more clustered as it takes into account how densely and how far away defect voxels are located from each other.

### Statistical analysis

As the first step, we focused on analyzing data that corresponds to the entire lung without differentiation between the lung sides. We compared outcomes of lung function tests (FEV_1_, FVC, FEV_1_/FVC, TLC, RV/TLC, LCI_2.5_) and MP-MRI parameters applied to the lung as a whole (VDP, QDP, DDI_V_, DDI_Q_, VQD_match_) between groups (healthy controls, small CDH, large CDH) using one-way ANOVA and corresponding post-hoc analysis (details given in the online supplement (OLS)). Age as a potential confounder did not differ significantly between the three groups and was therefore not implemented as a covariate in the final model.

Furthermore, we assessed differences in MP-MRI outcomes (VDP, QDP, DDI_V_, DDI_Q,_ and VQD_match_) between the affected (CDH-) and non-affected lung side. We tested whether these side differences varied between the groups (healthy controls, small CDH, large CDH) using a two-way repeated measures ANOVA with interaction between group and lung side and corresponding post hoc analysis (details given in the OLS). When quantifying VDP and QDP per lung side, we assessed the allocation of DPs to the right or left lung: the number of voxels with impaired ventilation or perfusion, respectively, per side was related to the number of voxels of the whole lung.

To investigate possible associations between lung function outcomes and functional MRI parameters, Spearman’s correlation coefficients (ρ) were calculated. We used the Benjamini-Hochberg procedure to correct for multiple comparisons.

Analysis was conducted in R version 4.1.2 [49]. A *p* value < 0.05 was considered statistically significant. Figures were produced using ggplot2 [50].

## Results

### Study population

We included thirteen patients with CDH of whom seven had a small and six a large initial defect size. The mean (range) age at the study visit was 9.9 years (4.7 to 13.6) in the small CDH group and 10.9 years (9.4 to 13.0) in the large CDH group. None of the patients included had received ECMO therapy or invasive ventilation until the age of 30 days. The control group consisting of thirteen subjects was matched for sex and age (mean 10.5 years, range 5.7 to 15.0). Details on demographic and clinical characteristics are shown in Table 1 and Supplemental Table S1.

**Table 1:** Study population characteristics

	Small CDH^1^	Large CDH^2^	Healthy controls
*n*	7	6	13
Females/males, *n* (%)	3/4 (42.9/57.1)	3/3 (50/50)	6/7 (46.2/53.8)
Age at study visit	9.89 ± 3.02	10.89 ± 1.43	10.48 ± 2.51
Weight at study visit (kg)	31.44 ± 8.79	27.55 ± 4.14	36.55 ± 10.44
z score	−0.45 ± 0.71	−1.74 ± 0.85	−0.14 ± 1.08
Height at study visit (cm)	138.71 ± 16.35	137.00 ± 10.28	140.23 ± 14.58
z score	−0.17 ± 0.78	−1.25 ± 1.14	−0.61 ± 0.80
BMI at study visit	16.05 ± 1.46	14.63 ± 0.90	18.28 ± 2.87
z score	−0.56 ± 0.83	−1.48 ± 0.75	0.24 ± 1.00
Side of hernia right/left, *n* (%)	1/6 (14.3/85.7)	1/5 (16.7/83.3)	NA
Time point of diagnosis, *n* (%)			
Prenatal	2 (28.6)	4 (66.6)	NA
< 24 h postnatal	1 (14.3)	2 (33.3)	NA
> 24 h postnatal	4 (57.1)	0 (0)	NA
Fetal tracheal plug, *n* (%)	0 (0)	2 (33.3)	NA
Age at surgery (days)	85.86 ± 171.34	2.17 ± 0.98	NA
Repair patch/muscle flap, *n* (%)		3/3 (50/50)	NA
Gestational age (weeks)	38.19± 1.75	37.36 ± 2.68	NA
Vaginal delivery/caesearian, *n* (%)	5/2 (57.1/28.6)	0/6 (0/100.0)	NA
Birth weight (g)	3344 ± 772	2909 ± 661	NA
Birth height (cm)	46.8 ± 7.4	48.62 ± 3.04	NA

Data are given as absolute counts (%) or mean ± SD

^1^ Defined as having received a primary closure of the diaphragmatic defect

^2^ Defined as having required a hernia repair with a patch or a muscle flap

*CDH*: congenital diaphragmatic hernia; *BMI*: body mass index; *NA*: non-applicable

### Overall assessment of lung function and MP-MRI outcomes

#### Spirometry, body plethysmography, and MBW

**Fig. 1:**
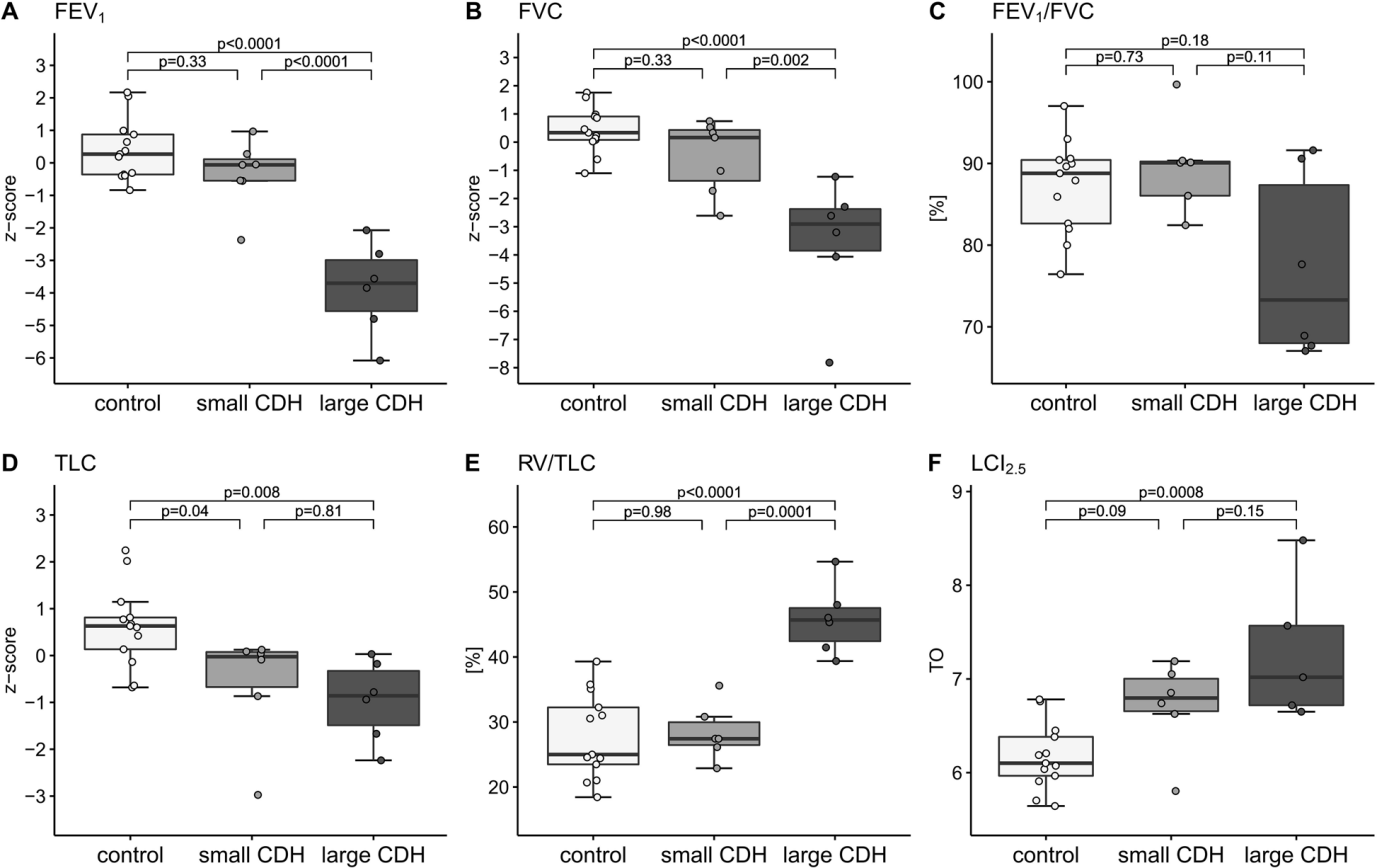
Lung function in healthy controls and patients with small and large CDH. Individual values in (**A**) FEV_1_ (z-score), (**B**) FVC (z-score), (**C**) FEV_1_/FVC (%), (**D**) TLC (z-score), (**E**) RV/ TLC (%), (**F**) LCI_2.5_ (TO). The group level is presented as grey boxplots (median, lower, and upper quartile, whiskers extending to at most 1.5*interquartile range). *p* values are the results of post hoc analysis of one-way ANOVA and are corrected for multiple testing. small CDH: congenital diaphragmatic hernia with primary closure; large CDH: congenital diaphragmatic hernia with patch or flap repair; FEV_1_: forced expiratory volume in 1 second; FVC: forced vital capacity; TLC: total lung capacity; RV: residual volume; LCI_2.5_: Lung clearance index, measured at classical end of nitrogen multiple-breath washout (2.5% of the normalized nitrogen starting concentration); TO: turnover (raw unit of LCI)

In patients with a large CDH, FEV_1_ z-score, and FVC z-score were significantly reduced in the post hoc analysis compared with healthy controls and patients with a small CDH, but FEV_1_/FVC was not. TLC z-score was significantly reduced in patients with CDH only compared with healthy controls. Moreover, in the post hoc analysis, patients with a large CDH had significantly higher (worse) RV/TLC and LCI_2.5_ values than healthy controls and, regarding RV/TLC, also than patients with a small CDH. Detailed results are given in Table 2, Fig. 1, and Supplemental Table S2 (all *p*s of underlying one-way ANOVA < 0.0009, shown in the OLS, Supplemental Table S3).

**Table 2:** Lung function outcomes of healthy controls and patients with small and large CDH

Parameters	Healthy Control	Small CDH^1^	Large CDH^2^	Mean Difference (95% CI_adj_)	*p* value_adj_
Spirometry	*n *= 13	*n *= 7	*n *= 6		
FEV_1_ (z-score)	0.40 ± 0.93	−0.33 ± 1.04		−0.73 (−2.01 to 0.54)	0.33
	0.40 ± 0.93		−3.86 ± 1.43	−4.26** (−5.61 to −2.92)	< 0.0001
		−0.33 ± 1.04	−3.86 ± 1.43	−3.53** (−5.04 to −2.01)	< 0.0001
FVC (z-score)	0.43 ± 0.80	−0.51 ± 1.29		−0.94 (−2.57 to 0.68)	0.33
	0.43 ± 0.80		−3.54 ± 2.30	−3.97** (−5.68 to −2.26)	< 0.0001
		−0.51 ± 1.21	−3.54 ± 2.30	−3.03** (−4.95 to −1.09)	0.002
FEV_1_/FVC (%)	87.26 ± 5.67	89.25 ± 5.45		1.99 (−4.86 to 8.84)	0.73
	87.26 ± 5.67		77.25 ± 11.39	−10.01 (−24.95 to 4.94)	0.18
			77.25 ± 11.39	−12.00 (−27.02 to 3.02)	0.11
Body plethysmography	*n *= 13	*n *= 6	*n *= 6		
TLC (z-score)	0.62 ± 0.87	−0.61 ± 1.21		−1.23* (−2.42 to −0.04)	0.04
	0.62 ± 0.87		−0.96 ± 0.87	−1.58** (−2.77 to −0.39)	0.008
		−0.61 ± 1.21	−0.96 ± 0.87	−0.35 (−1.74 to 1.04)	0.81
RV/TLC (%)	27.81 ± 6.58	28.38 ± 4.36		0.57 (−6.71 to 7.84)	0.98
	27.81 ± 6.58		45.82 ± 5.36	18.01** (10.73 to 25.28)	< 0.0001
		28.38 ± 4.36	45.82 ± 5.36	17.44** (8.92 to 25.95)	0.0001
N_2_MBW	*n *= 13	*n *= 6	*n *= 5		
LCI_2.5_ (TO)	6.17 ± 0.35	6.71 ± 0.49		0.54 (−0.06 to 1.15)	0.09
	6.17± 0.35		7.29 ± 0.76	1.12** (0.47 to 1.76)	0.0008
		6.71 ± 0.49	7.29 ± 0.76	0.58 (−0.17 to 1.32)	0.15
MP-MRI	*n *= 13	*n *= 7	*n *= 6		
VDP (%)	15.53 ± 4.55	15.03 ± 1.49		−0.50 (−4.07 to 3.07)	0.93
	15.53 ± 4.55		24.12 ± 3.46	8.59** (3.58 to 13.60)	0.002
		15.03 ± 1.49	24.12 ± 3.46	9.09** (4.54 to 13.64)	0.002
QDP (%)	14.53 ± 2.91	14.41 ± 4.17		−0.12 (−3.97 to 3.73)	> 0.99
	14.53 ± 2.91		31.75 ± 2.88	17.22** (13.16 to 21.27)	< 0.0001
		14.41 ± 4.17	31.75 ± 2.88	17.34** (12.76 to 21.90)	< 0.0001
DDI_V_ (arb. unit)	1.06 ± 0.62	1.53 ± 1.38		0.47 (−0.43 to 0.87)	0.49
	1.06 ± 0.62		2.51 ± 0.48	1.45** (0.82 to 2.13)	0.002
		1.53 ± 1.38	2.51 ± 0.48	0.98 (−0.05 to 1.98)	0.08
DDI_Q_ (arb. unit)	0.82 ± 0.50	1.58 ± 1.92		0.76 (−0.23 to 0.72)	0.21
	0.82 ± 0.50		5.49 ± 1.58	4.67** (2.94 to 6.29)	0.0002
		1.58 ± 1.92	5.49 ± 1.58	3.91** (1.94 to 6.13)	0.01
VQD_match_ (%)	2.05 ± 1.89	2.31 ± 2.39		0.26 (−2.52 to 3.05)	0.97
	2.05 ± 1.89		12.02 ± 3.24	9.97** (7.04 to 12.91)	< 0.0001
		2.31 ± 2.39	12.02 ± 3.24	9.71** (6.40 to 13.02)	< 0.0001

^*^ *p* < .05, ** *p* < .01

^1^ defined as having received a primary closure of the diaphragmatic defect

^2^ defined as having required a hernia repair with a patch or a muscle flap

Lung function parameters and functional MP-MRI parameters are given as z-scores or absolute values respectively, presented as mean ± standard deviation and compared by post hoc analysis of one-way ANOVA. Adjustment of CI and *p* values for multiple testing using Tukey (FEV_1_, FVC, RV/TLC, LCI_2.5_, QDP, VQD_match_), Games-Howell (FEV_1_/FVC, VDP) and Benjamini & Hochberg (DDI_V_, DDI_Q_) approaches

*CDH*: congenital diaphragmatic hernia; *CI*: confidence interval; adj: adjusted; *FEV*_*1*_: forced expiratory volume in 1 second; *FVC*: forced vital capacity; *RV*: residual volume; *TLC*: total lung capacity; *LCI*_*2.5*_: Lung clearance index, measured at classical end of nitrogen multiple-breath washout (N_2_MBW) (2.5% of the normalized nitrogen starting concentration); TO: lung turnover (raw unit of LCI); *MP-MRI*: Matrix-pencil decomposition magnetic resonance imaging; *VDP*: percentage of the lung volume with impaired fractional ventilation; *QDP*: percentage of lung volume with impaired relative perfusion; *DDI*_*V*_: defect distribution index of ventilation; *DDI*_*Q*_: defect distribution index of perfusion; *VQD*_*match*_: matched defect in perfusion and ventilation (in percent)

#### MP-MRI

**Fig. 2:**
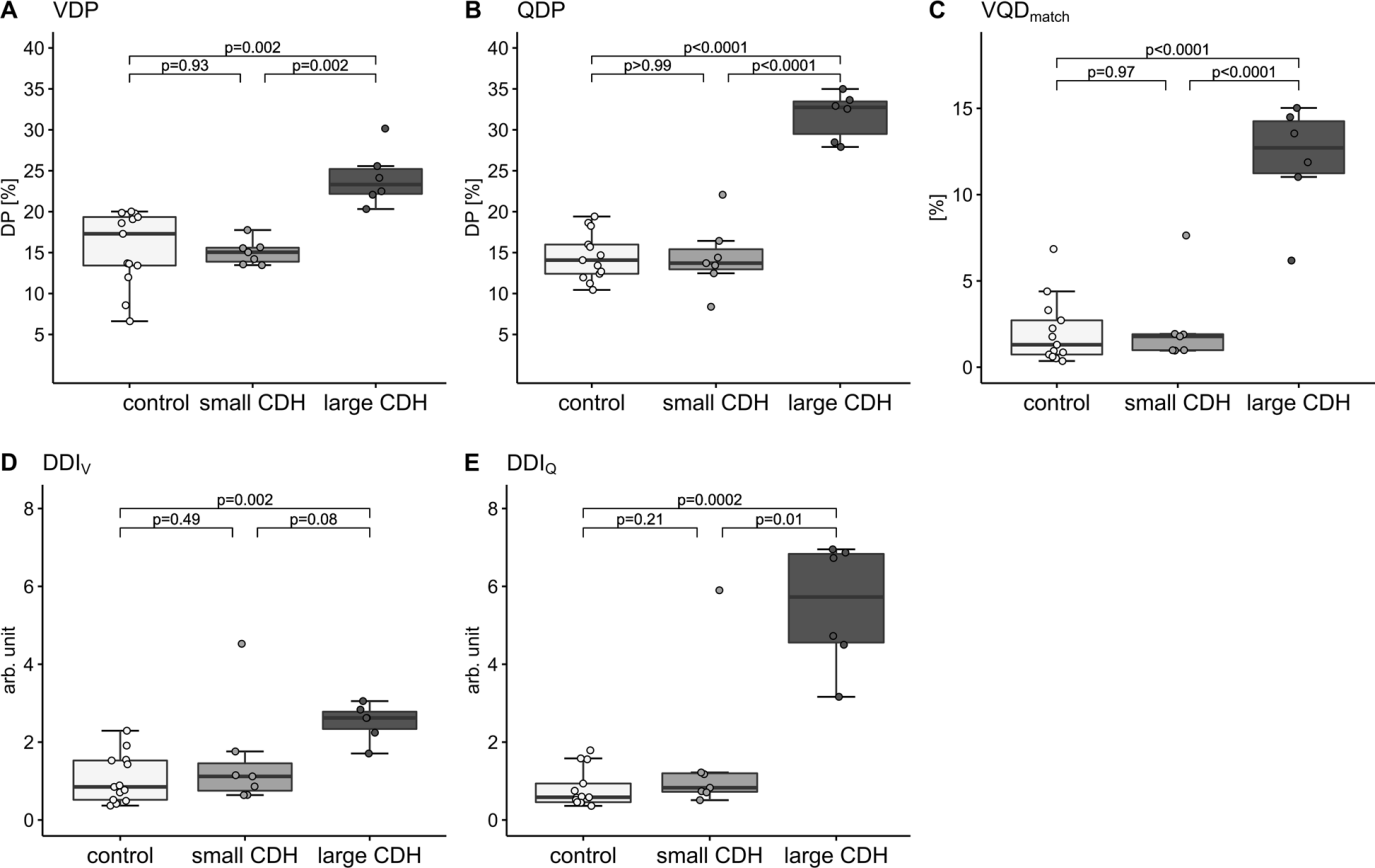
MP-MRI parameters in healthy controls and patients with small and large CDH. Individual values in (**A**) VDP (%), (**B**) QDP (%), (**C**) VQD_match_ (%), (**D**) DDI_V_ (arb. unit), and (**E**) DDI_Q_ (arb. unit). The group level is presented as grey boxplots (median, lower, and upper quartile, whiskers extending to at most 1.5*interquartile range). *p* values are results of post-hoc analysis of one-way ANOVA and corrected for multiple testing. MP-MRI: Matrix pencil decomposition magnetic resonance imaging; small CDH: congenital diaphragmatic hernia with primary closure; large CDH: congenital diaphragmatic hernia with patch or flap repair; VDP: percentage of the lung volume with impaired fractional ventilation; QDP: percentage of the lung volume with impaired relative perfusion; VQD_match_: matched defect in perfusion and ventilation (in percent); DDI_V_: defect distribution index of ventilation; DDI_Q_: defect distribution index of perfusion; DP: defect percentage

For each MP-MRI outcome, post hoc analysis showed a significant functional impairment in patients with a large CDH compared to healthy controls and patients with a small CDH, respectively. DDI_V_ was only significant between the large CDH group and healthy controls. Detailed results are given in Table 2, Fig. 2, and Supplemental Table S2 (all p's of underlying one-way ANOVA < 0.007, shown in the OLS, Supplemental Table S3). Additional regression analysis to adjust for orthopedic sequelae (scoliosis, pectus excavatum) in patients with large CDH was performed, and overall results did not change.

### Lung side-specific assessment of MP-MRI outcomes

**Fig. 3:**
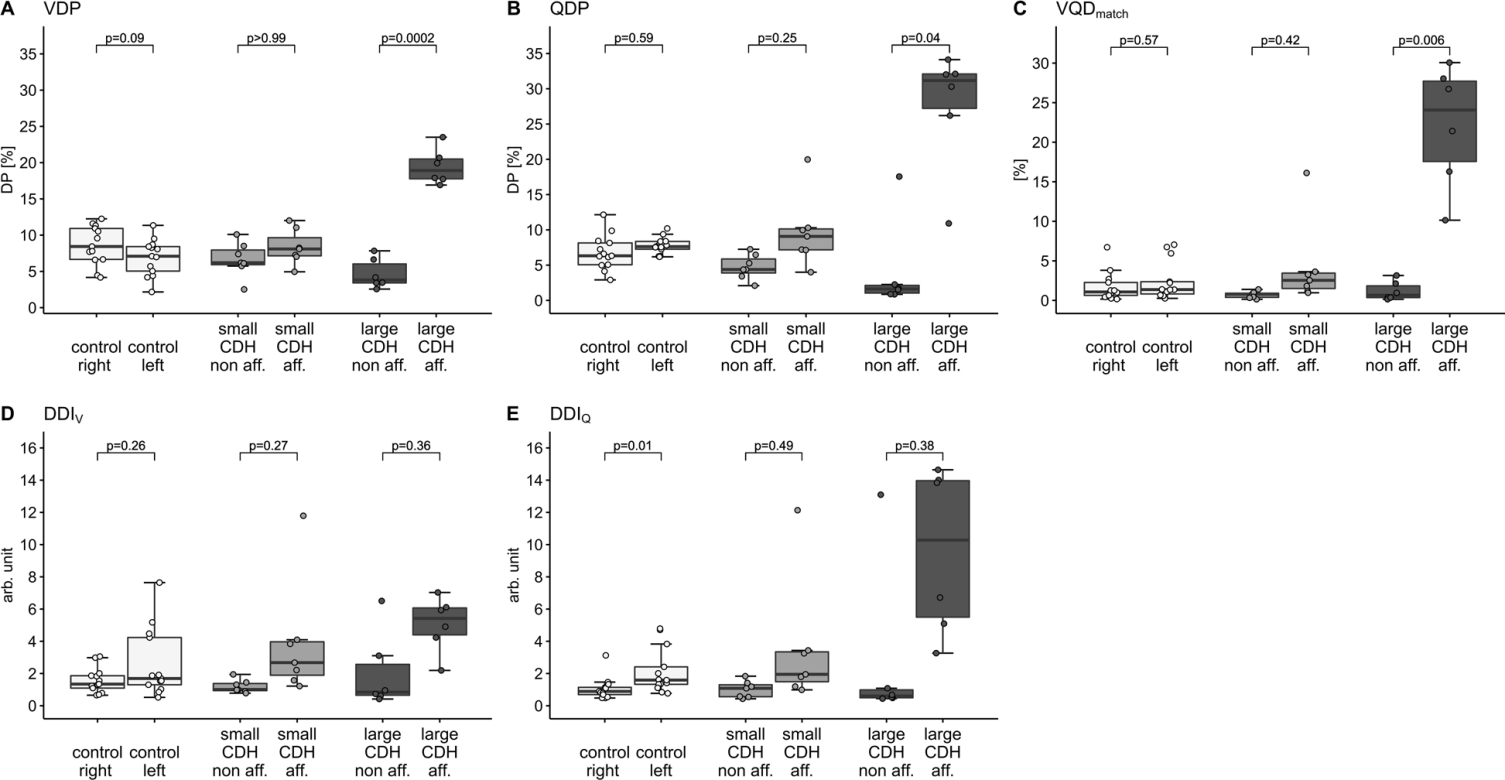
MP-MRI parameters in healthy controls and patients with small and large CDH, separated by lung side. Individual values for the ipsilateral lung side (affected) and the contralateral lung side (non-affected) in (**A**) VDP (%), (**B**) QDP (%), (**C**) VQD_match_ (%), (**D**) DDI_V_ (arb. unit) and (**E**) DDI_Q_ (arb. unit). In healthy controls, the left lung side was compared to the right lung side. The group level is presented as grey boxplots (median, lower, and upper quartile, whiskers extending to at most 1.5*interquartile range). *p* values are the results of post hoc analysis of two-way repeated measures ANOVA and corrected for multiple testing. MP-MRI: Matrix pencil decomposition magnetic resonance imaging; small CDH: congenital diaphragmatic hernia with primary closure; large CDH: congenital diaphragmatic hernia with patch or flap repair; VDP: percentage of the lung volume with impaired fractional ventilation; QDP: percentage of the lung volume with impaired relative perfusion; VQD_match_: matched defect in perfusion and ventilation (in percent); DDI_V_: defect distribution index of ventilation; DDI_Q_: defect distribution index of perfusion; DP: defect percentage; non aff.: non-affected, contralateral lung side; aff.: affected, ipsilateral lung side

**Fig. 4:**
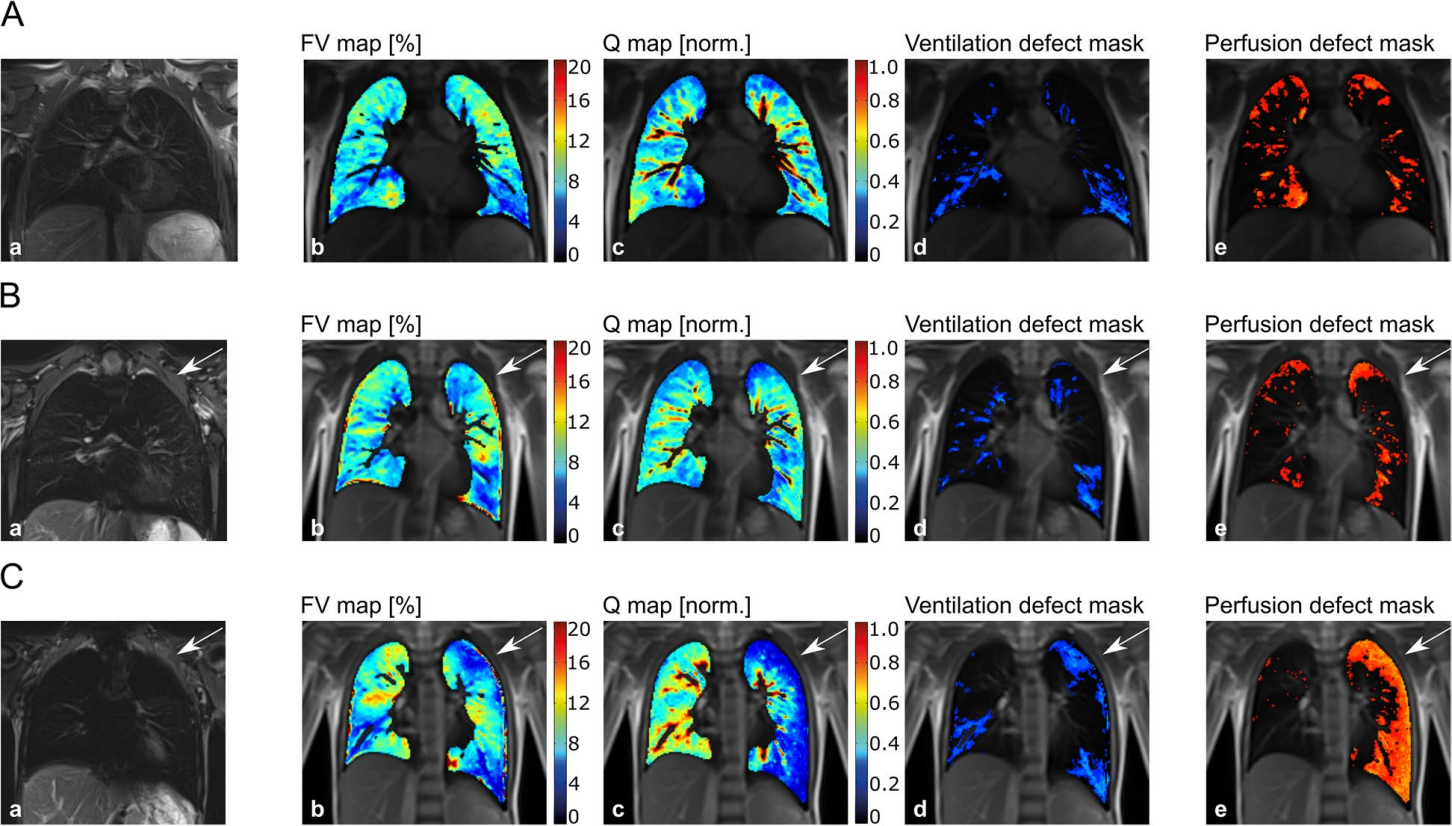
Example of MP-MRI images in (**A**) a healthy control, (**B**) a patient with a small CDH, and (**C**) a patient with a large CDH. **A:** 11.9-year-old, healthy boy (VDP_left_ (%) = 8.42, VDP_right_ (%) = 10.92, QDP_left_ (%) = 6.18, QDP_right_ (%) = 7.24). **B:** 8.7-year-old, female patient with small CDH (primary closure of the diaphragmatic defect) on the anatomically left side, marked by white arrow (VDP_affected_ (%) = 8.26, VDP_non-affected_ (%) = 7.39, QDP_affected_ (%) = 9.97, QDP_non-affected_ (%) = 6.47). **C:** 9.9-year-old, female patient with large CDH (flap repair of the diaphragmatic defect) on the anatomically left side, marked by white arrow (VDP_affected_ (%) = 17.74, VDP_non-affected_ (%) = 7.84, QDP_affected_ (%) = 32.11, QDP_non-affected_ (%) = 1.53). For each case study, morphological images are given in (a). Overlaid on morphological images are: fractional ventilation maps (b), relative perfusion maps (c), and masks representing areas with impaired ventilation and impaired perfusion (d, e). On the heat maps, a change of colour range towards dark blue indicates severe impairment of lung function. Further individual outcome values are provided in Supplemental Table S6. CDH: congenital diaphragmatic hernia; MP-MRI: Matrix pencil decomposition magnetic resonance imaging; VDP: percentage of the lung volume with impaired fractional ventilation; QDP: percentage of lung volume with impaired relative perfusion; non aff.: non-affected, contralateral lung side; aff.: affected, ipsilateral lung side

Differences in VDP, QDP, and VQD_match_ between the two lung sides varied among the groups. A significant impairment of ventilation and perfusion of the CDH-affected lung side compared to the contralateral side was found only in the large CDH group (mean difference): VDP (%) 14.80, *p*_adj _= 0.0002; QDP (%) 23.50, *p*_adj _= 0.04 and VQD_match_ (%) 20.93, *p*_adj _= 0.006. Detailed results are shown in Table 3, Fig. 3, Fig. 4, and Supplemental Table S4-S6 (all *p*s of underlying two-way repeated measures ANOVA < 0.0001, presented in the OLS, Supplemental Table S5). Both patients who had received a fetal tracheal plug were part of the large CDH group and their outcomes (both, overall and side-specific) did not differ from the other patients with a large CDH.

**Table 3:** MP-MRI outcomes according to lung side in healthy controls and patients with small and large CDH

Parameters	Group	Non-affected side^a^	Affected side^b^	Mean Difference (95% CI_adj_)	*p* value_adj_
VDP (%)	Control	8.62 ± 2.67	6.91 ± 2.52	−1.70 (−3.64 to 0.23)	0.09
	Small CDH^1^	6.65 ± 2.39	8.38 ± 2.42	1.73 (−3.96 to 7.41)	> 0.99
	Large CDH^2^	4.68 ± 2.08	19.44 ± 2.45	14.80** (10.50 to 19.00)	0.0002
QDP (%)	Control	6.79 ± 2.45	7.74 ± 1.18	0.95 (−0.99 to 2.90)	0.59
	Small CDH	4.75 ± 1.76	9.66 ± 5.03	4.91 (−2.88 to 12.70)	0.25
	Large CDH	4.13 ± 6.60	27.61 ± 8.60	23.50* (1.75 to 45.20)	0.04
DDI_V_ (arb. unit)	Control	1.57 ± 0.78	2.60 ± 2.12	1.03 (−0.50 to 2.56)	0.26
	Small CDH	1.20 ± 0.40	3.92 ± 3.63	2.72 (−1.75 to 7.19)	0.27
	Large CDH	2.06 ± 2.39	5.07 ± 1.71	3.01 (−2.66 to 8.68)	0.36
DDI_Q_ (arb. unit)	Control	1.06 ± 0.69	2.14 ± 1.40	1.08* (0.25 to 1.91)	0.01
	Small CDH	1.01 ± 0.52	3.53 ± 3.91	2.52 (−2.71 to 7.76)	0.49
	Large CDH	2.72 ± 5.09	9.59 ± 5.13	6.88 (−6.39 to 20.10)	0.38
	Control	1.72 ± 1.83	2.45 ± 2.44	0.73 (−0.73 to 2.18)	0.57
	Small CDH	0.70 ± 0.43	4.22 ± 5.34	3.52 (−3.28 to 10.30)	0.42
	Large CDH	1.18 ± 1.20	22.11 ± 7.71	20.93** (8.40 to 33.50)	0.006

^*^ *p* < .05, ** *p* < .01

^a^ right lung side in healthy controls

^b^ left lung side in healthy controls

^1^ defined as having received a primary closure of the diaphragmatic defect

^2^ defined as having required a hernia repair with a patch or a muscle flap

Lung function parameters and MP-MRI parameters are given as absolute values, presented as mean ± standard deviation and compared by post hoc analysis of two-way repeated measures ANOVA. Adjustment of CI and *p* values for multiple testing using Bonferroni correction. n_control _= 13; n_small CDH_=7; n_large CDH _= 6

*MP-MRI*: Matrix-pencil decomposition magnetic resonance imaging; *CDH*: congenital diaphragmatic hernia; *CI*: confidence interval; adj: adjusted; *VDP*: percentage of the lung volume with impaired fractional ventilation; *QDP*: percentage of lung volume with impaired relative perfusion; *DDI*_*V*_: defect distribution index of ventilation; *DDI*_*Q*_: defect distribution index of perfusion; *VQD*_*matc*h_: matched defect in perfusion and ventilation (in percent)

### Association of lung function and MP-MRI outcomes

**Fig. 5:**
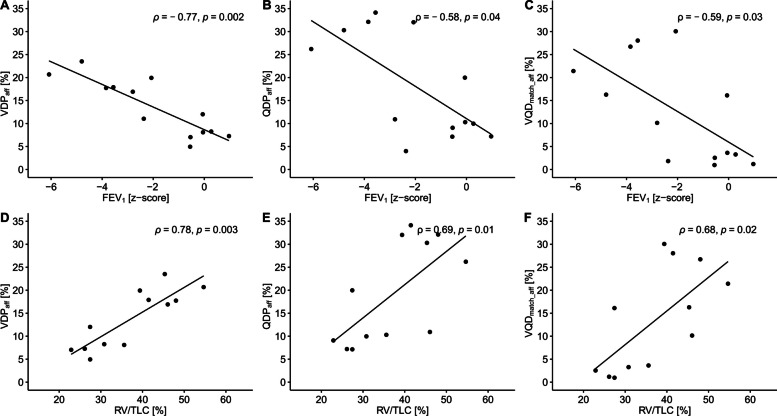
Association of lung function and MP-MRI outcomes of the lung side affected in patients with CDH. Individual values plotted by (**A**) FEV_1_ (z-score) and VDP (%), (**B**) FEV_1_ (z-score) and QDP (%), (**C**) FEV_1_ (z-score) and VQD_match_ (%), (**D**) RV/TLC and VDP (%), (**E**) RV/TLC and QDP (%), and (**F**) RV/TLC and VQD_match_ (%). Values of both, patients with a small CDH (primary closure of the diaphragmatic defect) and a large CDH (patch or flap repair of the diaphragmatic defect) are included. Lines represent simple linear regression (*ρ* = Spearman’s correlation coefficient). The Benjamini-Hochberg procedure has been applied to correct *p*-values for multiple comparisons. MP-MRI: Matrix pencil decomposition magnetic resonance imaging; CDH: congenital diaphragmatic hernia; FEV_1_: forced expiratory volume in 1 second; VDP: percentage of the lung volume with impaired fractional ventilation; QDP: percentage of lung volume with impaired relative perfusion; VQD_match_: matched defect in perfusion and ventilation (in percent); aff.: affected, ipsilateral lung side; RV: residual volume; TLC: total lung capacity

Within the group of patients with CDH, there was a significant association of FEV_1_ z-score and VDP, QDP, and VQD_match_ of the affected side. The same applies to RV/TLC. Furthermore, the FVC z-score and VDP of the affected side correlated significantly. Detailed results are shown in Fig. 5 and Supplemental Table S7.

## Discussion

### Summary

In this study, we used functional MP-MRI to follow up patients with CDH and thus assessed overall lung function but also lung side-specific information. Our results, based on a small sample, indicate the following: (i) patients with a large CDH had significantly reduced outcomes in both, lung function tests and functional MP-MRI parameters compared to healthy controls as well as to patients with a small CDH; (ii) overall deficits in patients with a large CDH were accompanied by a pronounced ventilation and perfusion impairment of the affected lung side; and (iii) ventilation and perfusion of the remaining lung tissue expanding into the thoracic cavity after a patch or flap repair does not function properly.

### Comparison with literature

We found lower FEV_1_ and elevated LCI_2.5_ values in patients with a large CDH defect; this is in accordance with previous studies showing that among patients with CDH, those with a patch repair or large initial defect size show impaired lung function in early and long-term follow-up [7, 13]. In patients with a large defect size of category C or D, FEV_1pp_ was decreased by 12.9% (95% CI: −21.7%, −4.2%) to 17.6% (95% CI: −24.9%, −10.3%) compared to patients with a smaller defect size of category A or B [13]. Similarly, in our study group, the FEV_1pp_ was 37.74% (95% CI: −55.18%, −20.29%) lower in patients with a large CDH compared to those with a small CDH. However, our results in FEV_1_/FVC, TLC, and RV/TLC in this patient group suggest a decrease in FEV_1_ due to reduced vital capacity upon hyperinflation rather than an obstruction. Further results reported in the literature are inconsistent, in some studies an effect of defect size on long-term lung function outcome or exercise capacity respectively was not detectable [12] or disappeared after one year of age [7].

Regarding functional MRI we found that relative impairment of ipsilateral ventilation and perfusion was highest in patients with a large CDH. In patients with a small CDH, there was no significant difference in ventilation or perfusion between the lung sides. These findings are in line with recent scintigraphy data that showed reduced ipsilateral perfusion [19] and a particularly frequent ipsilateral V/Q mismatch [18] in patients with patch repair compared to those with direct repair (i.e. large and small CDH). Also, previous studies using functional lung MRI (DCE, 3He) found less subdivided, enlarged alveoli, and reduced ipsilateral perfusion in patients with CDH [22–26, 51]. In our study, we show that in patients with a large CDH ipsilateral impairment of ventilation and perfusion is densely clustered (high DDI_V_ and DDI_Q_) and overlapping in accordance with the Euler-Liljestrand-effect (high VQD_match_). From a technical point of view, MP-MRI offers the advantage of assessing local ventilation and perfusion in parallel [28, 29, 38], whereas 3He or DCE MRIs are limited to one of these two functional aspects [52, 53]. However, in line with other non-contrast dynamic MRI approaches, MP-MRI does not allow measurement of absolute pulmonary air or blood flow [28, 29, 38]. Instead, low ventilation or perfusion relative to the remaining lung of the patient is detected and the amount of lung volume affected is quantified. This method has by now been established for the investigation of lung function in various diseases [34–37], even though direct comparability of outcome values is limited due to varying protocols for scan and analysis. In view of this, our study benefits from the direct inclusion of healthy controls and beyond, we ensured comprehensive assessment by using a multi-slice sequence covering the whole lung.

We also found a correlation between FEV_1_ z-score and ipsilateral ventilation as well as perfusion. So far, correlations between lung function outcomes and outcomes of functional imaging techniques have only been shown for ipsilateral perfusion parameters (FEV_1pp_ and pulmonary blood flow (PBF); FVC_pp_ and PBF [24]; FEF_25-75pp_ and scintigraphy perfusion [21]). Thus, our data show a function-function association between two very different methods: spirometry measuring airflow and volumes at the mouth and MP-MRI measuring ventilation impairment at a regional level.

### Strengths and limitations

The greatest strength of our study is the comprehensive follow-up assessment using lung function tests and functional MP-MRI of patients with CHD. We could compare both, spatially resolved perfusion and ventilation data of the lungs to outcomes of lung function tests. Further, we could investigate differences in long-term outcomes between patients with a small and a large CDH compared to healthy controls.

The main limitation of our study is the small sample size, and that the group of patients with a small CDH might represent a peculiar selection with a very mild course of disease. Thus, results should be seen as indicative only and generalized with caution. However, the findings on differences in lung-side specific impairment in the large CDH group are promising, as they are pathophysiologically reasonably explainable and even statistically significant using a conservative approach with adjusted p-values. The small side differences of the MP-MRI outcomes found in the healthy controls need to be further investigated. As the main conclusion of this manuscript is based on very pronounced side differences, we consider it to be unaffected by this issue.

### Physiological considerations and implications for clinical practice

Previous results on the long-term outcome of patients with CDH are to some extent contradictory. We were able to illustrate the strong contrast between the functionality of the ipsi- and the contralateral lung side in patients with reduced lung function. Thus, spatially resolved functional lung imaging provides a clear diagnostic advantage compared to regular lung function parameters such as spirometry and multiple-breath washout. While the assessed increased ipsilateral ventilation impairment might be partially affected by the measurement technique in combination with reduced diaphragmatic mobility, the result on perfusion, whose detection is clearly independent of diaphragmatic movement, confirms the results described. However, of course, a tissue biopsy would be desirable to confirm this conclusion. To date, it is unclear whether, in patients with a large initial defect size, the ipsilateral limitations are primarily due to altered fetal lung development or to decreased catch-up growth of the lung tissue including airways and vessels after patch or flap repair because of low diaphragmatic mobility [54]. In addition, other co-existing clinical conditions such as scoliosis or chest wall disorders might amplify respiratory restrictions. In this context, it might be particularly important to improve body posture and strengthen the respiratory muscles of the ipsilateral side by regular physiotherapy [15, 55]. Systematic long-term follow-up of patients with CDH taking lung side-specific assessment into account is indeed needed to clarify this issue. In this regard, MP-MRI is a promising tool since it is radiation free—therefore suited for regular (e.g., yearly) follow-up, does not require the administration of contrast agents, and is performed under free-breathing making it feasible already in infants and young children.

### Conclusions

In our study, we analyzed functional MP-MRI data on lung ventilation and perfusion in a small sample of patients with CDH. Our results confirm expected differences in functionality between the ipsi- and contralateral lung side in patients with large CDH. This indicates the importance of not only assessing overall lung function in patients with CDH but also performing a spatially resolved examination of the lung. Thus, MP-MRI is a promising tool for future follow-up or intervention studies in patients with CDH.

### Supplementary Information

Below is the link to the electronic supplementary material.

Supplementary file1 (PDF 318 KB)

## Data Availability

The data supporting the findings of this study are available from the corresponding author upon reasonable request

## Abbreviations

BMI: Body mass index
CDH: Congenital diaphragmatic hernia
CI: Confidence interval
CT: Computer tomography
DDI: Defect distribution index
DDI_Q_: Defect distribution index of perfusion
DDI_V_: Defect distribution index of ventilation
FEF25–75%: Forced expiratory flows at 25–75% of FVC
FEV_1_: Forced expiratory volume in one second
FRC_pleth_: Functional residual capacity derived from body plethysmography
FVC: Forced vital capacity
FVC: Forced vital capacity
IQR: Interquartile range
LCI: Lung clearance index
LLN: Lower limit of normal
MP: Matrix Pencil
MP-MRI: Matrix pencil decomposition magnetic resonance imaging
MRI: Magnetic resonance imaging
N_2_MBW: Nitrogen multiple-breath washout
OLS: Online supplement
pp: Percent predicted
Q: Perfusion
QDP: Perfusion defect percentage
RV/TLC: Residual volume over total lung capacity
S_acin_: Distal/ intra-acinar airways ventilation heterogeneity
S_cond_: Conducting airways ventilation heterogeneity
TO: Turnover
ULN: Upper limit of normal
V: Ventilation
VDP: Ventilation defect percentage
VQD_match_: matched defect in perfusion and ventilation (in percent)
ρ: Spearman’s correlation coefficient

## Declarations

### Guarantor

The scientific guarantor of this publication is Philipp Latzin, MD PhD; Division of Paediatric Respiratory Medicine and Allergology, Department of Paediatrics, Inselspital, Bern University Hospital, University of Bern, Switzerland. Mail: philipp.latzin@insel.ch

### Statistics and biometry

No complex statistical methods were necessary for this paper.

### Study subjects or cohorts overlap

None of our study subjects or cohorts have been previously reported

### Methodology

• retrospective

• observational

• performed at one institution
