# Supplementary material for: Long-term pulmonary outcome of children with congenital diaphragmatic hernia: functional lung MRI using matrix-pencil decomposition enables side-specific assessment of lung function
Source: Eur Radiol. 2023 Nov 20;34(6):3773–85. doi: 10.1007/s00330-023-10395-8 (PMC11166819; doi:10.1007/s00330-023-10395-8)
Supplement: Supplementary file 1 — Supplementary file1 (PDF 318 KB) [file 330_2023_10395_MOESM1_ESM.pdf]

Long-term pulmonary outcome of children with congenital diaphragmatic hernia: functional lung MRI using matrix-pencil decomposition enables side-specific assessment of lung function

Electronic Supplementary Material

Eur Radiol (2023) Streibel C, Willers CC, Bauman G et al.

Supplemental Methods

MP-MRI data acquisition and evaluation

MRI examinations including structural and functional scans were performed on a clinical 1.5T whole-body scanner (MAGNETOM Aera, Siemens Healthineers). Functional scans relied on a multi-slice 2D time-resolved ultra-fast balanced steady-state free precession (uf-bSSFP) pulse sequence (technical parameters: field-of-view (FOV) = 425 mm x 425 mm, 12-mm slice thickness, TE/TR = 0.67 ms/1.52 ms, flip angle a 65°, 2056 Hz/pixel bandwidth, 128 x 128 acquisition matrix (bicubic interpolation to 256 x 256), 150 coronal images, 110 ms per image, 3.33 images/s acquisition rate, 50 s total acquisition time per slice, parallel imaging GRAPPA factor 2) [1]. The uf-bSSFP pulse sequence applied used excitation pulses and gradient switching patterns of a conventional Cartesian bSSFP imaging scheme accompanied by partial echo readouts and ramp sampling techniques to shorten echo and repetition time, reduce motion and off-resonance artefacts and improve lung parenchyma signal [1]. Accordingly, the entire chest volume was covered from posterior to anterior with around 8 to 14 coronal slices and a voxel size of 3.3mm x 3.3mm x 12mm in supine position during free tidal breathing. At each slice location, 150 images were sequentially acquired during approximately 50 seconds with a frame rate of 3.3 images per second (110ms acquisition time per single image and 190ms interval between consecutive images). The time-resolved uf-bSSFP image series acquired was processed by elastic image registration to a fixed baseline image in mid respiratory state to compensate for respiratory motion [2]. Thus, the signal magnitude in each image was preserved, but lung structures (airways, vessels, thoracic walls, etc.) were aligned. On the registered images, the lung parenchyma was segmented automatically as described previously using a deep-learning algorithm [3].

Data (motion-corrected and segmented) were further processed with the matrix pencil (MP) algorithm derivated of Fourier decomposition [4]: Voxel-wise spectral analysis of the amplitudes of periodic lung parenchyma signal intensity modulations caused by respiration (frequency corresponding to respiratory rate) and pulsatile blood flow (frequency corresponding to pulse rate) was used to calculate quantitative ventilation and perfusion maps of the lung [4, 5]. Lung regions with the fractional ventilation or perfusion amplitude below 0.70 of the median of all pixels inside a local region of interest (segmented lung area on the corresponding coronal slice) were considered to show impaired fractional ventilation or impaired perfusion respectively [4, 5]. Main outcomes were ventilation defect percentage (VDP) and perfusion defect percentage (QDP), which equal the relative amount of lung volume with impaired fractional ventilation resp. relative perfusion [4, 5]. Homogeneity of defect distribution for ventilation and perfusion was assessed by the defect distribution index DDI (DDI_V and DDI_Q, resp.) [6]. The DDI increases with the defect areas being more clustered as it takes into account how densely and how far away defect voxels are located from each other.

Statistical Analysis

Regarding the comparison of the outcomes of the classic lung function tests (FEV_1, FVC, FEV_1/FVC, TLC, RV/TLC, $LCI_{2.5}$) and of the MP-MRI examination applied to the lung as a whole (VDP, QDP, DDI_V, DDI_Q, VQD_{match}) between the groups, we chose the method of analysis as required: According to data attributes, for FEV_1, FVC, TLC, RV/TLC, $LCI_{2.5}$, QDP and VQD_{match}, Fisher ANOVA, for FEV_1/FVC and VDP Welch ANOVA and for DDI_V and DDI_Q Kruskal-Wallis ANOVA was used. Accordingly, post-hoc analysis was calculated with Student t-test, Welch t-test and Mann-Whitney-U-test and per parameter corrected for multiple testing using Tukey, Games-Howell and Benjamini & Hochberg approaches, respectively.

Age as a potential confounder did not differ significantly between the three groups (tested using ANCOVA) and was therefore not implemented as a covariate in the final model.

Further, we assessed differences in MP-MRI outcomes (VDP, QDP, DDI_V, DDI_Q and VQD_{match}) between the affected (CDH-) and non-affected lung side. We tested whether these side differences varied between the groups (healthy controls, small CDH, large CDH) using a two-way repeated measures ANOVA including lung side as within-subject factor variable, group as between-subject factor variable and the interaction term between lung side and group. Per MP-MRI outcome parameter, post-hoc analysis was performed to assess side differences (affected vs. non-affected) in each group (healthy controls, small CDH, large CDH) using paired t-test comparisons with Bonferroni-correction for multiple testing.

Supplemental Tables

Supplemental Table S1. Study population characteristics

	**Small CDH[1]**	**Large CDH[2]**	**Healthy controls**
ECMO treatment, n (%)	0 (0)	0 (0)	NA
Invasive ventilation at age of 30 days, n (%)	0 (0)	0 (0)	NA
Duration of invasive ventilation in total (days)	2.43 ± 1.99	11.6 ± 6.31	NA
Duration hospitalization (days)	17.57 ± 6.65	133.6 ± 115.6	NA
at intensive care unit	4.71 ± 2.87	57.25 ± 59.73	NA
Scoliosis at study visit, n (%)	0 (0)	4 (66.7)	NA
Pectus exc. at study visit, n (%)	1 (14.3)	4 (66.7)	NA
Gastroesopageal reflux[3] at study visit, n (%)	0 (0)	2 (33.3)	NA

Data are given as absolute counts (%) or mean ± SD.

[1] defined as having received a primary closure of the diaphragmatic defect

[2] defined as having required a hernia repair with a patch or a muscle flap

[3] diagnosis based on clinical symptoms

CDH: congenital diaphragmatic hernia; ECMO: extracorporeal membrane oxygenation; pectus exc: pectus excavatus; NA: non applicable.

Supplemental Table S2. Lung function outcomes of healthy controls and patients with small and large CDH.

Parameters	Healthy Control	Small CDH[1]	Large CDH[2]	Mean Difference (95% CI_{adj})	p-value$_{adj}$	p-value$_{unadj}$
Spirometry	n=13	n=7	n=6			
FEV_1 (z-score)	0.40 ± 0.93	-0.33 ± 1.04		-0.73 (-2.01 to 0.54)	0.33	0.16
	0.40 ± 0.93		-3.86 ± 1.43	**-4.26**** (-5.61 to -2.92)	<0.0001	<0.0001
		-0.33 ± 1.04	-3.86 ± 1.43	**-3.53**** (-5.04 to -2.01)	<0.0001	<0.0001
FVC (z-score)	0.43 ± 0.80	-0.51 ± 1.29		-0.94 (-2.57 to 0.68)	0.33	0.16
	0.43 ± 0.80		-3.54 ± 2.30	**-3.97**** (-5.68 to -2.26)	<0.0001	<0.0001
		-0.51 ± 1.21	-3.54 ± 2.30	**-3.03**** (-4.95 to -1.09)	0.002	0.0007
FEV_1/FVC (%)	87.26 ± 5.67	89.25 ± 5.45		1.99 (-4.86 to 8.84)	0.73	0.46
	87.26 ± 5.67		77.25 ± 11.39	-10.01 (-24.95 to 4.94)	0.18	0.08
			77.25 ± 11.39	-12.00 (-27.02 to 3.02)	0.11	0.05
Body plethysmography	n=13	n=6	n=6			
TLC (z-score)	0.62 ± 0.87	-0.61 ± 1.21		**-1.23*** (-2.42 to -0.04)	0.04	0.02
	0.62 ± 0.87		-0.96 ± 0.87	**-1.58**** (-2.77 to -0.39)	0.008	0.003
		-0.61 ± 1.21	-0.96 ± 0.87	-0.35 (-1.74 to 1.04)	0.81	0.53
RV/TLC (%)	27.81 ± 6.58	28.38 ± 4.36		0.57 (-6.71 to 7.84)	0.98	0.85
	27.81 ± 6.58		45.82 ± 5.36	**18.01**** (10.73 to 25.28)	<0.0001	<0.0001
		28.38 ± 4.36	45.82 ± 5.36	**17.44**** (8.92 to 25.95)	0.0001	<0.0001
N_2MBW	n=13	n=6	n=5			

$LCI_{2.5}$ (TO)	6.17 ± 0.35	6.71 ± 0.49		0.54 (-0.06 to 1.15)	0.09	0.04
	6.17± 0.35		7.29 ± 0.76	**1.12**** (0.47 to 1.76)	0.0008	0.0003
		6.71 ± 0.49	7.29 ± 0.76	0.58 (-0.17 to 1.32)	0.15	0.06
MP-MRI	n=13	n=7	n=6			
VDP (%)	15.53 ± 4.55	15.03 ± 1.49		-0.50 (-4.07 to 3.07)	0.93	0.72
	15.53 ± 4.55		24.12 ± 3.46	**8.59**** (3.58 to 13.60)	0.002	0.0006
		15.03 ± 1.49	24.12 ± 3.46	**9.09**** (4.54 to 13.64)	0.002	0.0007
QDP (%)	14.53 ± 2.91	14.41 ± 4.17		-0.12 (-3.97 to 3.73)	>0.99	0.94
	14.53 ± 2.91		31.75 ± 2.88	**17.22**** (13.16 to 21.27)	<0.0001	<0.0001
		14.41 ± 4.17	31.75 ± 2.88	**17.34**** (12.76 to 21.90)	<0.0001	<0.0001
DDI_V (arb. unit)	1.06 ± 0.62	1.53 ± 1.38		0.47 (-0.43 to 0.87)	0.49	0.49
	1.06 ± 0.62		2.51 ± 0.48	**1.45**** (0.82 to 2.13)	0.002	0.0005
		1.53 ± 1.38	2.51 ± 0.48	0.98 (-0.05 to 1.98)	0.08	0.05
DDI_Q (arb. unit)	0.82 ± 0.50	1.58 ± 1.92		0.76 (-0.23 to 0.72)	0.21	0.21
	0.82 ± 0.50		5.49 ± 1.58	**4.67**** (2.94 to 6.29)	0.0002	0.0001
		1.58 ± 1.92	5.49 ± 1.58	**3.91**** (1.94 to 6.13)	0.01	0.008
VQD_{match} (%)	2.05 ± 1.89	2.31 ± 2.39		0.26 (-2.52 to 3.05)	0.97	0.81
	2.05 ± 1.89		12.02 ± 3.24	**9.97**** (7.04 to 12.91)	<0.0001	<0.0001
		2.31 ± 2.39	12.02 ± 3.24	**9.71**** (6.40 to 13.02)	<0.0001	<0.0001

* p < .05, ** p < .01
[1] defined as having received a primary closure of the diaphragmatic defect
[2] defined as having required a hernia repair with a patch or a muscle flap

Lung function parameters and functional MP-MRI parameters are given as z-scores or absolute values respectively, presented as mean ± standard deviation and compared by post-hoc analysis of one-way ANOVA. Adjustment of CI and p-values for multiple testing using Tukey (FEV_1, FVC, RV/TLC, $LCI_{2.5}$, QDP, VQD_{match}), Games-Howell (FEV_1/FVC, VDP) and Benjamini & Hochberg (DDI_V, DDI_Q) approaches.

Abbreviations: CDH: congenital diaphragmatic hernia; CI: confidence interval; adj: adjusted; FEV_1: forced expiratory volume in 1 second; FVC: forced vital capacity; RV: residual volume; TLC: total lung capacity; $LCI_{2.5}$: Lung clearance index, measured at classical end of nitrogen multiple-breath washout (N_2MBW) (2.5% of the normalized nitrogen starting concentration); TO: lung turnover (raw unit of LCI); MP-MRI: Matrix-pencil decomposition magnetic resonance imaging; VDP: percentage of the lung volume with impaired fractional ventilation; QDP: percentage of lung volume with impaired relative perfusion; DDI_V: defect distribution index of ventilation; DDI_Q: defect distribution index of perfusion; VQD_{match}: matched defect in perfusion and ventilation (in percent).

Supplemental Table S3. Whole lung function outcomes compared between healthy controls and patients with small and large CDH, results of one-way ANOVA.

Parameter	Statistical Analysis	Results
FEV_1	Fisher ANOVA	$F(2,23)=32.48$, $p<0.0001$
FVC	Fisher ANOVA	$F(2,23)=17.00$, $p<0.0001$
FEV_1/FVC	Welch ANOVA	$F(2,1.26)=2.61$, $p=0.12$
TLC	Fisher ANOVA	$F(2,22)=6.89$, $p=0.005$
RV/TLC	Fisher ANOVA	$F(2,22)=21.05$, $p<0.0001$
$LCI_{2.5}$	Fisher ANOVA	$F(2,21)=10.01$, $p=0.0009$
VDP	Welch ANOVA	$F(2,11.69)=17.07$, $p=0.0003$
QDP	Fisher ANOVA	$F(2,23)=63.89$, $p<0.0001$
DDI_V	Kruskal-Wallis ANOVA	$H(2)=9.89$, $p=0.007$
DDI_Q	Kruskal-Wallis ANOVA	$H(2)=13.31$, $p=0.001$
VQD_{match}	Fisher ANOVA	$F(2,23)=40.02$, $p<0.0001$

CDH: congenital diaphragmatic hernia; FEV_1: forced expiratory volume in 1 second; FVC: forced vital capacity; RV: residual volume; TLC: total lung capacity; $LCI_{2.5}$: Lung clearance index, measured at classical end of nitrogen multiple-breath washout (N_2MBW) (2.5% of the normalized nitrogen starting concentration); MP-MRI: Matrix pencil decomposition magnetic resonance imaging; VDP: percentage of the lung volume with impaired fractional ventilation; QDP: percentage of lung volume with impaired relative perfusion; DDI_V: defect distribution index of ventilation; DDI_Q: defect distribution index of perfusion; VQD_{match}: combined ventilation and perfusion defect percentage.

Supplemental Table S4. MP-MRI outcomes according to lung side in healthy controls and patients with small and large CDH.

Parameters	Group	Non-affected side[a]	Affected side[b]	Mean Difference (95% CI_{adj})	$p\text{-value}_{adj}$	$p\text{-value}_{unadj}$
**VDP** (%)	control	8.62 ± 2.67	6.91 ± 2.52	-1.70 (-3.64 to 0.23)	0.09	0.03
	small CDH[1]	6.65 ± 2.39	8.38 ± 2.42	1.73 (-3.96 to 7.41)	>0.99	0.36
	large CDH[2]	4.68 ± 2.08	19.44 ± 2.45	**14.80**** (10.50 to 19.00)	0.0002	<0.0001
**QDP** (%)	control	6.79 ± 2.45	7.74 ± 1.18	0.95 (-0.99 to 2.90)	0.59	0.20
	small CDH	4.75 ± 1.76	9.66 ± 5.03	4.91 (-2.88 to 12.70)	0.25	0.08
	large CDH	4.13 ± 6.60	27.61 ± 8.60	**23.50*** (1.75 to 45.20)	0.04	0.01
**DDI_V** (arb. unit)	control	1.57 ± 0.78	2.60 ± 2.12	1.03 (-0.50 to 2.56)	0.26	0.09
	small CDH	1.20 ± 0.40	3.92 ± 3.63	2.72 (-1.75 to 7.19)	0.27	0.09
	large CDH	2.06 ± 2.39	5.07 ± 1.71	3.01 (-2.66 to 8.68)	0.36	0.12
**DDI_Q** (arb. unit)	control	1.06 ± 0.69	2.14 ± 1.40	**1.08*** (0.25 to 1.91)	0.01	0.004
	small CDH	1.01 ± 0.52	3.53 ± 3.91	2.52 (-2.71 to 7.76)	0.49	0.16
	large CDH	2.72 ± 5.09	9.59 ± 5.13	6.88 (-6.39 to 20.10)	0.38	0.13
**VQD_{match}** (%)	control	1.72 ± 1.83	2.45 ± 2.44	0.73 (-0.73 to 2.18)	0.57	0.19
	small CDH	0.70 ± 0.43	4.22 ± 5.34	3.52 (-3.28 to 10.30)	0.42	0.14
	large CDH	1.18 ± 1.20	22.11 ± 7.71	**20.93**** (8.40 to 33.50)	0.006	0.002

* p < .05, ** p < .01
[a] right lung side in healthy controls
[b] left lung side in healthy controls
[1] defined as having received a primary closure of the diaphragmatic defect

[2] defined as having required a hernia repair with a patch or a muscle flap

Lung function parameters and MP-MRI parameters are given as absolute values, presented as mean ± standard deviation and compared by post-hoc analysis of two-way repeated measures ANOVA. Adjustment of CI and p-values for multiple testing using Bonferroni correction. $n_{control}$=13; $n_{small\ CDH}$=7; $n_{large\ CDH}$=6.

Abbreviations: MP-MRI: Matrix-pencil decomposition magnetic resonance imaging; CDH: congenital diaphragmatic hernia; CI: confidence interval; adj: adjusted; VDP: percentage of the lung volume with impaired fractional ventilation; QDP: percentage of lung volume with impaired relative perfusion; DDI_V: defect distribution index of ventilation; DDI_Q: defect distribution index of perfusion; VQD_{match}: matched defect in perfusion and ventilation (in percent).

Supplemental Table S5. Differences in MP-MRI outcomes between the affected (CDH-) and non-affected lung side, variation between the groups (healthy controls, small CDH, large CDH). Results of two-way repeated measures ANOVA.

**Parameter**		
**VDP**	(Intercept)	F(1,23)= 553.29, p<0.0001
	lung side	F(1,23)=53.16, p<0.0001
	group	F(2,23)=12.72, p=0.0002
	lung side*group	F(2,23)=53.03, p<0.0001
**QDP**	(Intercept)	F(1,23)= 886.04, p<0.0001
	lung side	F(1,23)=35.44, p<0.0001
	group	F(2,23)=63.89, p<0.0001
	lung side*group	F(2,23)=16.97, p<0.0001
**DDI_V**	(Intercept)	F(1,23)= 95.89, p<0.0001
	lung side	F(1,23)=13.45, p=0.001
	group	F(2,23)=2.47, p=0.11
	lung side*group	F(2,23)=1.25, p=0.31
**DDI_Q**	(Intercept)	F(1,23)= 106.64, p<0.0001
	lung side	F(1,23)=12.03, p=0.002
	group	F(2,23)=18.11, p<0.0001
	lung side*group	F(2,23)=2.94, p=0.07
**VQD_{match}**	(Intercept)	F(1,23)= 108.13, p<0.0001
	lung side	F(1,23)=62.93, p<0.0001
	group	F(2,23)=32.75 p<0.0001
	lung side*group	F(2,23)=33.40, p<0.0001

CDH: congenital diaphragmatic hernia; MP-MRI: Matrix pencil decomposition magnetic resonance imaging; VDP: percentage of the lung volume with impaired fractional ventilation; QDP: percentage of lung volume with impaired relative perfusion; DDI_V: defect distribution index of ventilation; DDI_Q: defect distribution index of perfusion; VQD_{match}: combined ventilation and perfusion defect percentage.

Supplemental Table S6. Outcome values of case studies presented in Figure 4.

Parameters	A. Healthy control	B. Patient with small CDH[1]	C. Patient with large CDH[2]
Spirometry			
FEV_1 (z-score)	0.64	0.27	-3.85
FVC (z-score)	1.59	0.53	-2.61
FEV_1/FVC (%)	76.44	86.05	67.69
Body plethysmography			
TLC (z-score)	1.15	0.04	0.03
RV/TLC (%)	18.40	30.80	48.00
N_2MBW			
$LCI_{2.5}$ (TO)	6.07	5.80	7.57
MP-MRI[3]			
$VDP_{affected}$ (%)	8.42	8.26	17.74
$VDP_{non-affected}$ (%)	10.92	7.39	7.84
$QDP_{affected}$ (%)	6.18	9.97	32.11
$QDP_{non-affected}$ (%)	7.24	6.47	1.53
$DDI_{V\_affected}$ (arb. unit)	4.23	3.84	4.91
$DDI_{V\_non-affected}$ (arb. unit)	2.01	0.98	3.12
$DDI_{Q\_affected}$ (arb. unit)	1.11	3.25	13.84
$DDI_{Q\_non-affected}$ (arb. unit)	0.69	1.41	0.68
$VQD_{match\_affected}$ (%)	2.22	3.27	26.72
$VQD_{match\_non-affected}$ (%)	2.27	0.82	0.95

[1] having received a primary closure of the diaphragmatic defect

[2] having required a hernia repair with a muscle flap

[3] in healthy control: left lung side assigned to "affected", right lung side assigned to "non-affected"

CDH: congenital diaphragmatic hernia; FEV_1: forced expiratory volume in 1 second; FVC: forced vital capacity; RV: residual volume; TLC: total lung capacity; $LCI_{2.5}$: Lung clearance index, measured at classical end of nitrogen multiple-breath washout (N_2MBW) (2.5% of the normalized nitrogen starting concentration); TO: lung turnover (raw unit of LCI); MP-MRI: Matrix-pencil decomposition magnetic resonance imaging; VDP: percentage of the lung volume with impaired fractional ventilation; QDP: percentage of lung volume with impaired relative perfusion; DDI_V: defect distribution index of ventilation; DDI_Q: defect distribution index of perfusion; VQD_{match}: matched defect in perfusion and ventilation (in percent).

Supplemental Table S7. Association between lung function and functional MRI outcomes in patients with CDH.

Association of parameters given as Spearman´s Rho. The Benjamini-Hochberg procedure has been applied for correction for multiple comparisons.

	**FEV$_1$**	**FVC**	**TLC**	**RV/ TLC**	**LCI$_{2.5}$**	**VDP$_{aff}$**	**VDP$_{non\text{-}aff}$**	**QDP$_{aff}$**	**QDP$_{non\text{-}aff}$**	**DDI$_{V_aff}$**	**DDI$_{V_non\text{-}aff}$**	**DDI$_{Q_aff}$**	**DDI$_{Q_non\text{-}aff}$**	**VQD$_{match_aff}$**
**FVC**	0.95**													
**TLC**	0.62**	0.72**												
**RV/TLC**	-0.83**	-0.79**	-0.25											
**LCI$_{2.5}$**	-0.53^{t}	-0.55^{t}	-0.21	0.32										
**VDP$_{aff}$**	-0.77**	-0.67*	-0.25	0.78**	0.46									
**VDP$_{non\text{-}aff}$**	0.25	0.27	-0.08	-0.43	-0.02	-0.49^{t}								
**QDP$_{aff}$**	-0.58*	-0.41	-0.13	0.69*	0.18	0.8**	-0.18							
**QDP$_{non\text{-}aff}$**	0.45	0.26	0.08	-0.37	-0.24	-0.65*	0.24	-0.81**						
**DDI$_{V_aff}$**	-0.31	-0.18	0.32	0.42	0.13	0.51^{t}	-0.58*	0.58**	-0.44					
**DDI$_{V_non\text{-}aff}$**	<0.01	0.08	-0.01	-0.22	0.40	-0.18	0.77**	-0.04	0.04	-0.3				
**DDI$_{Q_aff}$**	-0.51^{t}	-0.34	-0.03	0.57^{t}	0.23	0.76**	-0.31	0.95**	-0.87**	0.71**	-0.15			
**DDI$_{Q_non\text{-}aff}$**	0.04	-0.08	-0.01	0.17	-0.37	-0.38	0.08	-0.55^{t}	0.75**	0.19	-0.18	-0.62*		
**VQD$_{match_aff}$**	-0.59*	-0.45	-0.11	0.68*	0.22	0.86**	-0.37	0.96**	-0.84**	0.64*	-0.23	0.97**	-0.55*	
**VQD$_{match_non\text{-}aff}$**	-0.10	-0.19	-0.29	-0.03	0.21	-0.08	0.60*	-0.04	0.44	-0.37	0.51^{t}	-0.26	0.19	-0.19

^{t} $p < 0.1$, * $p < 0.05$, ** $p < 0.01$; Number of patients included: for FEV$_1$ and FVC n=13, for TLC n=12, for LCI$_{2.5}$ n=12, for MP-MRI parameters n=13.

MP-MRI: Matrix pencil decomposition magnetic resonance imaging; FEV$_1$: forced expiratory volume in 1 second (z-score); FVC: forced vital capacity (z-score); TLC: total lung capacity (z-score); RV: residual volume; aff.: lung side affected of CDH; non-aff.: lung side non affected of CDH; LCI$_{2.5}$: Lung clearance index, measured at classical end of nitrogen multiple-breath washout (N_2MBW) (2.5% of the normalized nitrogen starting concentration) (turnovers); VDP: percentage of the lung volume with impaired fractional ventilation; (%); QDP: percentage of lung volume with impaired relative perfusion (%); DDI$_V$: defect distribution index of ventilation (arb. unit); DDI$_Q$: defect distribution index of perfusion (arb. unit); VQD$_{match}$: matched defect in perfusion and ventilation (%).

REFERENCES

1. Bauman G, O Pusterla, O Bieri (2016) Ultra-fast Steady-State Free Precession Pulse Sequence for Fourier Decomposition Pulmonary MRI. Magn Reson Med DOI: 10.1002/mrm.25697
2. Sandkühler R, C Jud, S Pezold, PC Cattin. *Adaptive Graph Diffusion Regularisation for Discontinuity Preserving Image Registration*. in *Biomedical Image Registration*. 2018. Cham: Springer International Publishing.
3. Willers C, G Bauman, S Andermatt et al (2021) The impact of segmentation on whole-lung functional MRI quantification: Repeatability and reproducibility from multiple human observers and an artificial neural network. Magn Reson Med DOI: 10.1002/mrm.28476
4. Bauman G, O Bieri (2017) Matrix pencil decomposition of time-resolved proton MRI for robust and improved assessment of pulmonary ventilation and perfusion. Magn Reson Med DOI: 10.1002/mrm.26096
5. Nyilas S, G Bauman, G Sommer et al (2017) Functional magnetic resonance imaging as a new additional modality in the assessment of primary ciliary dyskinesia. European Respiratory Journal DOI: https://dx.doi.org/10.1183/1393003.congress-2017.PA3343
6. Valk A, C Willers, K Shahim et al (2021) Defect distribution index: A novel metric for functional lung MRI in cystic fibrosis. Magn Reson Med DOI: 10.1002/mrm.28947
